# Effects of Grape Seed Proanthocyanidin Extract on Vascular Endothelial Function in Participants with Prehypertension: A Randomized, Double-Blind, Placebo-Controlled Study

**DOI:** 10.3390/nu11122844

**Published:** 2019-11-20

**Authors:** Tamami Odai, Masakazu Terauchi, Kiyoko Kato, Asuka Hirose, Naoyuki Miyasaka

**Affiliations:** 1 Department of Obstetrics and Gynecology, Tokyo Medical and Dental University, 1-5-45 Yushima, Bunkyo-ku, Tokyo 113-8510, Japan; odycrm@tmd.ac.jp (T.O.); a-kacrm@tmd.ac.jp (A.H.); n.miyasaka.gyne@tmd.ac.jp (N.M.); 2 Department of Women’s Health, Tokyo Medical and Dental University, 1-5-45 Yushima, Bunkyo-ku, Tokyo 113-8510, Japan; kiyo.crm@tmd.ac.jp

**Keywords:** flow-mediated dilation, hypertension, vascular elasticity, vascular stiffness, polyphenols, flavonoids

## Abstract

This study aimed to investigate the effects of grape seed proanthocyanidin extract (GSPE) on blood pressure and vascular endothelial function in middle-aged Japanese adults with prehypertension. We conducted a randomized, double-blind, placebo-controlled study on 6 men and 24 women aged 40–64 years old. The participants were randomized to receive tablets containing either low-dose (200 mg/day) or high-dose (400 mg/day) GSPE, or placebo, for 12 weeks. Systolic and diastolic blood pressures (SBP and DBP, respectively), brachial flow-mediated dilation (FMD), and other cardiovascular parameters were measured before and after 4, 8, and 12 weeks of treatment. The mean SBP in the high-dose group significantly decreased by 13 mmHg after 12 weeks (*P* = 0.028), although FMD did not change. In an ad hoc analysis of non-smoking participants (*n* = 21), the mean SBP, DBP, stiffness parameter β, distensibility, incremental elastic modulus (Einc), and pulse wave velocity (PWV) also significantly improved in the high-dose group after 12 weeks. Changes in Einc and PWV from baseline to 12 weeks were significantly greater in the high-dose group than in the placebo group (Einc, *P* = 0.023; PWV, *P* = 0.03). GSPE consumption could help maintain vascular elasticity and normal blood pressure in this population.

## 1. Introduction

Hypertension is one of the major risk factors for cardiovascular diseases (CVDs), which are a leading cause of global mortality. According to the World Health Organization, increased blood pressure (BP) is estimated to contribute to 9.4 million deaths annually, accounting for approximately 16.5% of the total mortality, and the global prevalence of hypertension in adults aged 18 years old or older was approximately 22.3% in 2015 [1,2]. Over recent decades, endothelial function impairment has been shown to play a key role in the early stages of atherosclerosis [3,4], linking cardiovascular risk factors, such as hypertension, dyslipidemia, diabetes mellitus, and chronic smoking, to endothelial dysfunction [5,6]. Nitric oxide (NO), an endothelium-dependent relaxing factor, also plays a central role in BP control [7,8]. Therefore, the preservation of normal endothelial function and regulation of BP are crucial to preventing progression to CVDs.

In the presence of risk factors for CVDs, including hypertension, diabetes mellitus, dyslipidemia, and smoking, increased generation of reactive oxygen species (ROS) contributes to endothelial dysfunction [6,9,10]. For example, elevated ROS levels in hypertensive patients leads to reduced vascular bioavailability of NO [7,9]. Hence, antioxidants that improve oxidative stress status are expected to promote vascular health, thereby preventing CVDs. Several studies involving rodents and humans with hypertension or CVDs showed that antioxidants, including vitamins C and E, genistein, and polyphenols, enhanced vascular endothelial function and decreased BP via reduction in ROS levels [11,12,13,14,15,16,17]. Nevertheless, several meta-analyses of randomized trials reported that antioxidant supplements exhibit no protective effects against CVDs [18,19,20]. Proanthocyanidin, a class of polyphenols, has strong antioxidant properties [21,22]. The antioxidant activity of proanthocyanidin is superior to that of vitamins C and E, β-carotene, or monomeric flavanol, including (+)-catechin [21,22,23,24]. Furthermore, grape seed extracts containing 39–73% proanthocyanidin have also been shown to have strong antioxidant potency [21]. Recently, we have reported that consumption of grape seed proanthocyanidin extract (GSPE) for 8 weeks decreased BP in middle-aged Japanese women [25]. However, the mechanism by which GSPE decreases BP is yet to be elucidated. Flow-mediated dilation (FMD) is a noninvasive measure widely used as an index of endothelial function through the quantification of vascular response to NO release [26]. The present study aimed to investigate the effects of GSPE on BP and endothelial functions, as assessed by FMD, in middle-aged Japanese men and women with prehypertension.

## 2. Methods

### 2.1. Study Population

**Figure 1:**
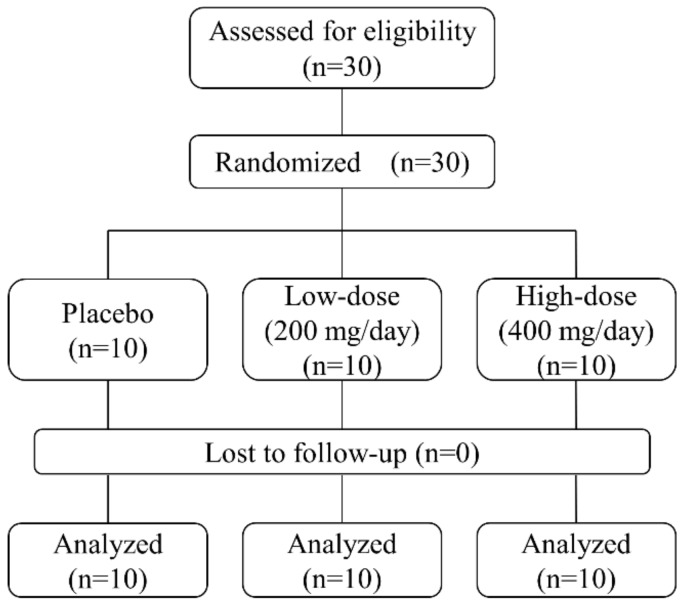
Study participant assignment.

We conducted a randomized, double-blind, placebo-controlled study at the Menopause Clinic of Tokyo Medical and Dental University Hospital. The study participants included prehypertensive Japanese men and women aged 40–64 years old and were recruited via a research support agency (Pacific Grove, Tokyo, Japan). Prehypertension was defined as systolic BP (SBP) of 130–139 mmHg and/or diastolic BP (DBP) of 85–89 mmHg, according to the 2014 diagnostic criteria of the Japanese Society of Hypertension. The study participants had been diagnosed with prehypertension in their most recent medical checkup. Those who had been treated for non-communicable diseases (e.g., hypertension, dyslipidemia, and diabetes) were excluded prior to study enrollment by the research support agency. The study sponsor (Kikkoman Biochemifa Company, Tokyo, Japan) randomly assigned the participants to the study groups and provided the GSPE tablets (Gravinol™). The participants were randomized to receive GSPE tablets that contained either low-dose (200 mg/day, *n* = 10) or high-dose (400 mg/day, *n* = 10) proanthocyanidin, or placebo (*n* = 10), for 12 weeks (Figure 1). Each group comprised 2 men and 8 women. Both investigators and participants were blinded to study group assignments. The GSPE tablets contained concentrated proanthocyanidin (~85%) and flavan-3-ol monomers such as catechin, epicatechin, and epicatechin gallate. The low- and high-dose GSPE and placebo tablets (Kikkoman Biochemifa Company, Tokyo, Japan) were indistinguishable in shape, weight, and color. The participants were instructed to take four tablets per day at any time of the day. Each tablet contained 100, 50, or 0 mg of GSPE for the high-dose, low-dose, and placebo groups, respectively. Participants underwent a series of examinations before and after 4, 8, and 12 weeks of treatment and were checked for adherence. We regarded a tablet intake of <75% as therapeutic noncompliance.

The study was conducted in accordance with the Declaration of Helsinki and the study protocol was reviewed and approved by the Tokyo Medical and Dental University Review Board (UMIN000030171). Written informed consent form was obtained from all participants.

### 2.2. Measurement

#### 2.2.1. Vascular Functions

FMD was defined as the maximum percentage increase in vessel diameter based on the resting vascular diameter, as measured using high-resolution ultrasonography (UNEXEF-38G; UNEX, Nagoya, Japan) for endothelial function assessment. First, the resting diameter of the right brachial artery was measured in the dorsal position after 10 minutes of rest, and a cuff placed around the forearm was subsequently inflated to a suprasystolic pressure (50 mmHg above SBP). After 5 minutes, the cuff was deflated and the baseline vascular diameter and dilation response were measured. FMD and FMDb represent the peak percentage increase in diameter above the resting and baseline diameters, respectively. Several studies have reported that the baseline diameter after cuff occlusion is smaller than the resting diameter depending on the resting vascular tone (referred to as low-flow-mediated constriction (L-FMC)), making FMDb a vascular marker comparable to FMD [27,28]. Furthermore, we evaluated vascular parameters simultaneously obtained by sequential analysis (Trend Plus™, UNEXEF-38G; UNEX, Nagoya, Japan). These parameters, including intima-media thickness, wall-to-lumen ratio, stiffness parameter β, compliance, distensibility, incremental elastic modulus (Einc), and pulse wave velocity (PWV), were automatically and concurrently calculated when FMD was measured. Brachial intima-media thickness and the wall thickness-to-vascular diameter ratio have been shown to be associated with the risk of CVDs and chronic heart failure, respectively [29,30]. Stiffness parameter β, compliance, distensibility, Einc, and PWV are widely recognized as vascular elasticity parameters associated with the risk of CVD development [31,32,33,34].

#### 2.2.2. Other Cardiovascular Risk Parameters

A vascular screening system (VS-1000; Fukuda Denshi, Tokyo, Japan) was used to evaluate SBP, DBP, heart rate, cardio-ankle vascular index, and ankle-brachial pressure index. Additionally, serum lipid profiles, including high- and low-density lipoprotein cholesterols, oxidized low-density lipoprotein cholesterol, triglyceride, and total cholesterol, were investigated. Blood examination was conducted in accordance with the guidelines on internal and external quality control defined by the Japanese Ministry of Health, Labour and Welfare. Participants were also evaluated for current smoking status (yes vs. no).

#### 2.2.3. Body Composition

The participants’ body composition, including height, weight, body mass index, fat mass, lean body mass, muscle mass, water mass, and basal metabolic rate, was measured using a body composition analyzer (MC190-EM; Tanita, Tokyo, Japan).

#### 2.2.4. Outcome Measures

FMD was the primary outcome measure in this study, and other cardiovascular risk parameters and body composition were the secondary outcome measures.

### 2.3. Statistical Analysis

Based on a previous study [25], we estimated a total sample size of 32, as calculated from an effect size of 5.5 mmHg, standard deviation of 4.0 mmHg, two-sided alpha of 0.05, and power of 0.8. Continuous variables are presented as the mean ± standard deviation. Differences along the time course and between the three groups were analyzed using the paired t-test, unpaired t-test, chi-square test, and one-way analysis of variance. *P* < 0.05 was considered statistically significant. All statistical analyses were performed using GraphPad Prism version 5.02 (GraphPad Software, San Diego, CA, USA) and JMP version 12 (SAS Institute Inc., Cary, NC, USA).

## 3. Results

The 6 men and 24 women who were enrolled in the study completed the 12-week study without side effects, and their self-reported adherence ranged from approximately 85% to 100%. Their average age was 53.7 ± 7.7 years. The three groups presented with similar baseline characteristics before treatment (Table 1). In some recruited participants who met the pre-defined criteria for prehypertension at the time of enrollment, the mean SBP and DBP at baseline were within the hypertensive range. FMD and FMDb did not change significantly after 12 weeks in any of the study groups. After 12 weeks of treatment, the mean SBP in the high-dose group significantly decreased. Furthermore, the mean stiffness parameter β, distensibility, and PWV improved after 8 and 12 weeks, and Einc decreased after 12 weeks of intervention in the high-dose group (Table S1). No significant changes in these parameters from baseline to 12 weeks were observed when compared with those in the placebo group (Table 1). In the low-dose group, there were no significant differences in any of the parameters of vascular function and cardiovascular risk, except for the mean body mass index, muscle mass, water mass, and basal metabolic rate, which significantly increased only at 12 weeks.

**Figure 2:**
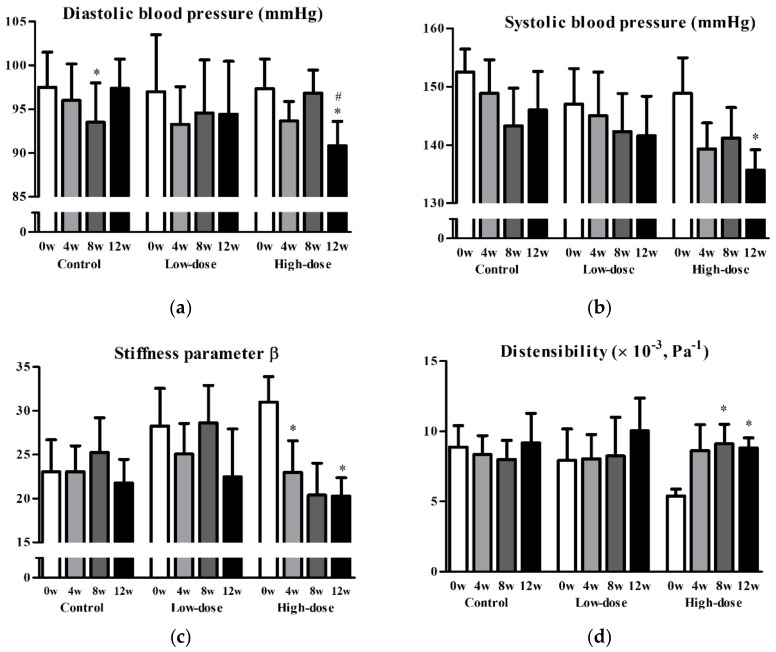

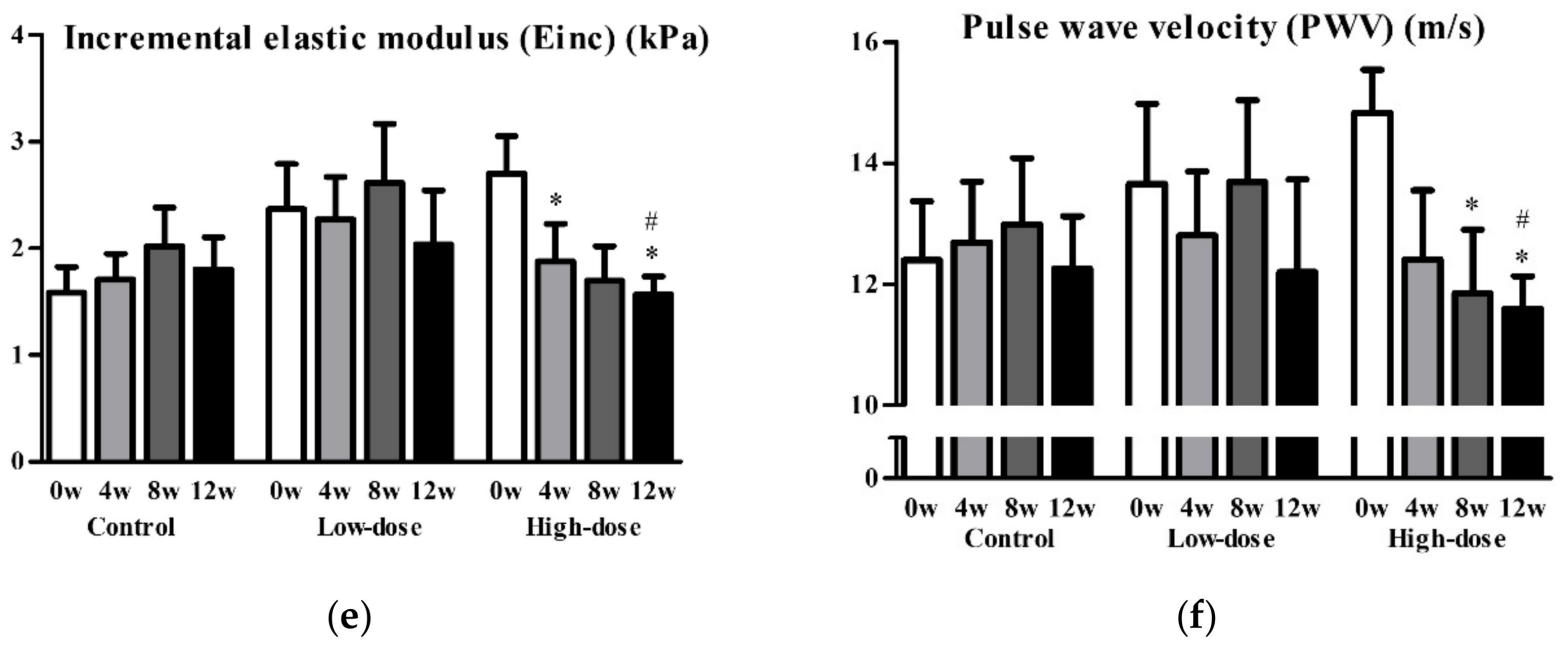
Diastolic blood pressure (**a**), systolic blood pressure (**b**), stiffness parameter β (**c**), distensibility (**d**), incremental elastic modules (**e**), and pulse wave velocity (**f**) before and after the intervention in non-smoking groups. Data are presented as mean and standard error. * *P* < 0.05 vs. baseline, paired t-test. # *P* < 0.05, change from baseline vs. placebo, unpaired t-test.

Next, we performed a post hoc analysis of non-smoking participants to assess the effects of GSPE on vascular functions, excluding 9 subjects who were exposed to strong oxidative stress. In 21 non-smoking participants, the placebo, low-dose, and high-dose groups comprised 8, 7, and 6 participants, respectively, with each group including one man. No statistically significant differences in any of the parameters at baseline were observed between the subgroups, except for the wall thickness-to-vascular diameter ratio (placebo, 0.092 ± 0.009; low-dose, 0.077 ± 0.020; high-dose, 0.073 ± 0.013; *P* = 0.033). The mean SBP and DBP were significantly reduced by 13.1 and 6.5 mmHg, respectively, in the high-dose group after 12 weeks of intervention (Figure 2a,b). Additionally, stiffness parameter β, distensibility, Einc, and PWV significantly improved in the high-dose group after 12 weeks (stiffness parameter β, 31.0 ± 7.1 to 20.3 ± 5.1, *P* = 0.039; distensibility, 5.4 ± 1.2 to 8.8 ± 1.8 (×10^−3^) Pa^−1^, *P* = 0.010; Einc, 2.7 ± 0.9 to 1.6 ± 0.4 kPa, *P* = 0.017; PWV, 14.8 ± 1.7 to 11.6 ± 1.3 m/s, *P* = 0.019) (Figure 2c–f). Changes in Einc and PWV from baseline to 12 weeks were significantly greater in the high-dose group than in the placebo group (high-dose vs. placebo: Einc, −1.13 ± 0.78 vs. 0.22 ± 1.08 kPa, *P* = 0.023; PWV, −3.2 ± 2.3 vs. −0.1 ± 2.3 m/s, *P* = 0.030) (Figure 2e,f). None of the parameters of vascular function and cardiovascular risk in the low-dose group were significantly different after the intervention, except for a reduction in high-density lipoprotein cholesterol (baseline, 73.4 ± 18.1 mg/dL; 12 weeks, 66.6 ± 18.9; *P* = 0.044). Nonetheless, there was no significant change in high-density lipoprotein cholesterol from baseline to 12 weeks when compared to that in the placebo group (*P* = 0.150).

## 4. Discussion

In this randomized, double-blind, placebo-controlled study, high-dose GSPE decreased BP in prehypertensive middle-aged Japanese men and women. GSPE improved vascular elasticity in non-smoking participants without affecting FMD.

Proanthocyanidin, often called “condensed tannin”, is an oligomer/polymer built from flavan-3-ol units and is common among plants, being particularly abundant in the fruits, seeds, nuts, and bark [22,35,36,37]. Proanthocyanidin has been demonstrated to possess several health-promoting and disease-preventing properties, such as antioxidant, anticancer, anti-inflammatory, and anti-atherosclerotic effects [22,36,38,39,40,41].

Several studies have reported the effects of grape polyphenols on endothelial function in humans, although their outcomes are difficult to compare because of the differences in purity, dosage, duration of administration, and background factors, such as age and cardiovascular risk. One randomized, double-blind, crossover study involving 24 men with metabolic syndrome showed that the administration of grape polyphenols for 30 days improved SBP and FMD, and changes in SBP were inversely correlated with NO production [42]. In another randomized crossover study that included 36 men and women at high vascular risk, FMD increased after 4 weeks of grape polyphenol treatment, although there were no effects on other cardiovascular parameters, such as BP, lipid profiles, clotting factors, and vascular elasticity [43]. Conversely, in a randomized, double-blind, crossover study of 35 healthy men, a significant effect of grape polyphenols on FMD was not observed after 2 weeks of intervention [44]. Additionally, Mellen et al. showed in a randomized, double-blind, crossover study of 50 adults at risk or with a history of CVDs, that muscadine grape seed increased resting brachial diameter, although FMD did not change significantly [45]. Their finding suggests that the increase in resting diameter reflecting baseline vascular relaxation did not lead to a further increase in FMD, as FMD is expressed as a change from the resting diameter, which appears to be supported by the demonstration of an inverse correlation between FMD and resting diameter [46].

There have been several studies on L-FMC, that is, vasoconstriction during cuff inflation. Although FMD provides information on endothelial vasomotor function in response to stimuli, it does not represent pre-existent vascular status. Gori et al. proposed that FMD is a response to the sudden increase in shear-stress, whereas, L-FMC reflects resting endothelium-dependent vascular tone [28]. It has been reported that L-FMC was regulated by several vasoactive substances, such as endothelin-1 (ET-1), endothelium-derived hyperpolarizing factor, and cyclooxygenase product [28,47]. L-FMC has also been shown to be affected by several other factors, it decreases in patients who smoke or have CVDs and increases with exercise [28,48,49]. Gori et al. claimed that low L-FMC are vascular risk factors, while high L-FMC reflects favorable endothelial health [50]. Harrison et al. also demonstrated that L-FMC was inversely correlated with PWV [51]. In the present study, the mean baseline vascular diameter was smaller than that of the resting diameter after 12 weeks of treatment and FMDb showed an increasing trend only in the high-dose group (Table S1). These findings could explain the improvement in vascular health with high-dose GSPE administration.

Endothelium largely contributes to the maintenance of vascular homeostasis through the production and secretion of physiologically active substances, such as relaxing and clotting factors, inflammatory cytokines, and their anti-factors. Although vascular endothelial homeostasis is maintained by a balance of several chemical mediators, FMD mainly reflects NO production accompanying increase in shear stress. Our findings that GSPE improved BP and vascular elasticity without affecting FMD indicate that the antioxidant effects of GSPE could regulate vascular tone, not through NO release, but by other endothelial responses, which resulted in BP reduction. One study on hypertensive rats that supports our results showed the positive association between ROS level and PWV, arterial wall thickness, and collagen deposition and the beneficial effects of antioxidants on arterial stiffness and remodeling [52], implying that the antioxidant capacity of GSPE could contribute to decreased ROS levels and improved vascular elasticity. Furthermore, several studies on the antioxidant effects of grape polyphenols on arteriosclerosis in vitro and in vivo reported that (i) grape seed polyphenols had potent anti-inflammatory properties via their inhibition of inflammatory cytokine production (e.g., interleukin-1β, prostaglandin E2, tumor necrosis factor-α, and C-reactive protein) [53,54]; (ii) grape flavanols hindered vascular smooth muscle cells from proliferating in vitro [55,56]; (iii) grape polyphenols inhibited platelet aggregation via suppression of platelet/endothelial cell adhesion molecule-1 activation in vitro [57,58]; and (iv) flavan-3-ols and procyanidins suppressed the activity of angiotensin-converting enzyme in vitro, which could reduce vasoconstriction and arteriosclerosis induced by angiotensin II [59]. Moreover, several studies have reported the antihypertensive effects of GSPE and its underlying mechanism in hypertensive rats. For instance, Quiñones et al. showed that GSPE increased prostaglandin F1α, a stable indicator of the production of prostaglandin I2, which is a vasodilator and inhibitor of platelet aggregation [60]. Liu et al. showed that GSPE decreased the expression of both ET-1, a known contributor to vasoconstriction and vascular remodeling, and transforming growth factor-β, which stimulates ET-1 expression and renin release [61,62]. Moreover, Huang et al. reported that GSPE reduced the release of ET-1, hampering the p38/c-jun N-terminal kinase mitogen-activated protein kinase pathway related to stress response [63]. Pons et al. showed that, in addition to reducing the ET-1 levels, GSPE downregulated the expression of nicotinamide adenine dinucleotide phosphate oxidase, a ROS generator [64]. Further studies are required to elucidate exactly how GSPE affects vascular health.

It is known that cigarette smoking is a strong inducer of free radicals, impairing endothelial function in a dose-dependent manner [65,66,67,68]. We failed to show significant changes in BP and vascular elasticity from baseline to 12 weeks between the placebo and high-dose groups among the participants including smokers, indicating that the inhibitory effects of cigarette smoking on vascular function could have been too potent for GSPE to overcome.

The present study has several limitations. Firstly, the sample size was small, particularly in a post hoc analysis, and the study duration was short. The study population consisted only of Japanese adults; therefore, it is difficult to extrapolate our current findings to a wider population. Moreover, we could not determine whether the effects of GSPE depended on sex differences due to the small population size. Secondly, the study participants were not administered with the pure polyphenol antioxidant, but with the grape seed extract with concentrated proanthocyanidin. Although the test product had relatively high purity (~85%), we could not exclude the potential effects of coexistent flavan-3-ol monomers or some unknown molecules. Thirdly, we did not evaluate plasma proanthocyanidin concentration and oxidative stress status. Finally, factors influencing vascular health, such as diet, exercise habits, and intake of other nutritional supplements, were not vigorously assessed. Despite these limitations, our study has several strengths; it was a randomized, double-blind, placebo-controlled study, and we evaluated various cardiovascular parameters and body composition indices concurrently with FMD. However, a large-scale randomized controlled trial seems necessary to verify our findings.

## 5. Conclusions

Twelve weeks of high-dose GSPE treatment in prehypertensive middle-aged Japanese men and women improved vascular elasticity and BP. GSPE consumption could ameliorate vascular stiffness and maintain normal BP in this population.

## Figures and Tables

**Table 1:** Parameters before and after 12 weeks of the intervention in each group.

	Placebo (*n* = 10)			Low Dose *(n* = 10)				High Dose (*n* = 10)				*P* ^c^
	0w	12w	*P* ^a^	0w	12w	*P* ^a^	P ^b^	0w	12w	*P* ^a^	*P* ^b^	
Age, years	55.5 (6.9)			53.5 (8.0)				52.2 (8.6)				0.644 ^d^
Smoker	2			3				4				0.621 ^e^
Vascular functions												
Resting vascular diameter, mm	3.82 (0.70)	3.69 (0.67)	0.182	3.68 (0.52)	3.75 (0.56)	0.492	0.163	3.85 (0.56)	3.64 (0.44)	0.236	0.764	0.809 ^d^
Flow-mediated dilation (FMD), %	4.2 (1.5)	4.3 (1.7)	0.262	5.4 (1.6)	4.5 (3.1)	0.349	0.362	4.6 (1.3)	3.4 (2.4)	0.162	0.189	0.187^d^
Baseline vascular diameter, mm	3.84 (0.69)	3.70 (0.70)	0.132	3.69 (0.54)	3.76 (0.55)	0.399	0.083	3.85 (0.58)	3.57 (0.46)	0.136	0.538	0.799 ^d^
Flow-mediated dilation from baseline (FMDb), %	3.6 (1.9)	4.2 (2.0)	0.451	5.4 (2.4)	4.3 (2.5)	0.290	0.347	4.6 (2.2)	5.6 (2.7)	0.422	0.722	0.208 ^d^
Intima-media thickness, mm	0.33 (0.08)	0.29 (0.08)	0.192	0.27 (0.04)	0.29 (0.06)	0.469	0.103	0.27 (0.05)	0.26 (0.04)	0.832	0.250	0.068 ^d^
Wall thickness-to-vascular diameter ratio	0.09 (0.02)	0.08 (0.02)	0.355	0.08 (0.02)	0.08 (0.02)	0.741	0.287	0.07 (0.02)	0.07 (0.01)	0.726	0.248	0.126 ^d^
Stiffness parameter β	23.1 (9.4)	19.8 (7.9)	0.301	25.5 (11.1)	21.7 (12.1)	0.340	0.866	28.0 (7.1)	20.6 (5.0)	0.025 ^*^	0.353	0.426 ^d^
Compliance (×10^-2^), mm^2^/Pa	10.2 (5.8)	10.3 (5.3)	0.953	8.9 (5.2)	10.7 (6.1)	0.355	0.580	7.4 (3.5)	8.9 (1.8)	0.239	0.662	0.503 ^d^
Distensibility (×10^-3^), Pa^-1^	8.8 (3.9)	10.0 (5.6)	0.510	8.4 (5.0)	9.6 (5.2)	0.463	0.859	6.1 (1.6)	8.6 (1.7)	0.004 ^**^	0.654	0.387 ^d^
Incremental elastic modulus (Einc), kPa	1.6 (0.6)	1.7 (0.8)	0.931	2.1 (1.0)	1.9 (1.1)	0.309	0.380	2.53 (0.9)	1.7 (0.4)	0.018 ^*^	0.243	0.162 ^d^
Pulse wave velocity (PWV), m/sec	12.3 (2.5)	11.6 (2.5)	0.417	12.9 (3.2)	12.2 (3.4)	0.515	0.896	14.1 (2.0)	11.7 (1.3)	0.009 ^**^	0.290	0.440 ^d^
Other cardiovascular parameters												
Systolic blood pressure (SBP), mmHg	150.1 (11.1)	144.0 (17.0)	0.201	144.0 (18.1)	142.5 (17.1)	0.706	0.411	148 (13.0)	135.0 (12.6)	0.028 ^*^	0.334	0.634 ^d^
Diastolic blood pressure (DBP), mmHg	98.4 (10.2)	97.1 (8.4)	0.489	95.9 (15.4)	94.3 (13.6)	0.637	0.991	96.0 (6.9)	89.9 (7.9)	0.062	0.150	0.858 ^d^
Heart rate, min^-1^	69.9 (12.4)	68.8 (9.4)	0.685	72.6 (9.4)	71.8 (11.4)	0.837	1.000	67.0 (11.3)	69.9 (13.9)	0.375	0.404	0.537 ^d^
Cardio-ankle vascular index	8.5 (0.7)	8.5 (0.7)	1.000	8.1 (1.3)	8.0 (1.1)	0.885	0.738	7.6 (1.1)	8.1 (0.9)	0.067	0.577	0.749 ^d^
Ankle-brachial pressure index	1.12 (0.05)	1.13 (0.08)	0.686	1.10 (0.06)	1.12 (0.06)	0.336	0.711	1.13 (0.08)	1.18 (0.06)	0.135	0.290	0.551 ^d^
High-density lipoprotein cholesterol, mg/dL	63.3 (19.4)	61.7 (10.0)	0.647	70.6 (16.0)	66.8 (15.7)	0.244	0.416	77.9 (14.7)	75.6 (11.7)	0.375	0.573	0.171 ^d^
Low-density lipoprotein cholesterol, mg/dL	111.0 (25.4)	115.6 (25.7)	0.301	128.2 (37.1)	126.1 (35.9)	0.694	0.426	110.8 (32.5)	112.1 (21.2)	0.835	0.913	0.391 ^d^
Oxidized low-density lipoprotein cholesterol, U/L	116.6 (47.2)	97.7 (38.2)	0.262	115.8 (45.6)	110.1 (48.4)	0.705	0.608	106.2 (34.8)	113.3 (29.5)	0.507	0.171	0.834 ^d^
Triglyceride, mg/dL	99.9 (55.2)	114.9 (46.8)	0.234	87.9 (55.3)	149.5 (125.6)	0.096	0.262	103.5 (71.7)	121.3 (74.4)	0.116	0.917	0.838 ^d^
Total cholesterol, mg/dL	192.5 (26.5)	196.2 (22.8)	0.459	219.1 (31.8)	219.1 (32.1)	1.000	0.543	207.5 (41.1)	207.4 (33.1)	0.987	0.700	0.226 ^d^
Body composition												
Height, cm	158.7 (11.6)	158.8 (11.4)	0.986	159.1 (8.5)	158.9 (8.4)	0.963	0.154	159.6 (10.5)	159.4 (10.4)	0.961	0.117	0.982 ^d^
Weight, kg	59.4 (13.1)	59.2 (13.1)	0.524	58.9 (8.6)	59.7 (8.6)	0.076	0.060	57.6 (13.7)	57.5 (12.6)	0.844	0.558	0.945 ^d^
Body mass index, kg/m^2^	23.4 (3.4)	23.3 (3.5)	0.311	23.4 (3.7)	23.7 (3.8)	0.050 ^*^	0.024 ^*^	22.5 (3.9)	22.5 (3.7)	0.751	0.299	0.586 ^d^
Fat mass, kg	17.5 (7.1)	17.1 (7.4)	0.175	17.9 (7.2)	17.9 (7.6)	0.969	0.340	15.4 (7.9)	15.5 (7.4)	0.744	0.842	0.722 ^d^
Lean body mass, kg	41.9 (9.7)	42.1 (9.4)	0.050	41.0 (5.1)	41.8 (5.7)	0.744	0.219	42.2 (9.2)	42.0 (8.9)	0.444	0.242	0.944 ^d^
Muscle mass, kg	39.6 (9.2)	39.8 (8.9)	0.331	38.7 (4.9)	39.5 (5.4)	0.047 ^*^	0.210	39.9 (8.8)	39.7 (8.5)	0.961	0.228	0.882 ^d^
Water mass, kg	30.0 (5.8)	30.1 (5.6)	0.512	29.5 (3.0)	30.6 (3.7)	0.035 ^*^	0.508	29.7 (5.2)	29.6 (5.0)	0.771	0.537	0.942 ^d^
Basal metabolic rate, MJ/day	5.04 (1.06)	5.05 (1.04)	0.574	4.95 (0.53)	5.04 (0.57)	0.027 ^*^	0.085	5.05 (1.04)	5.02 (0.98)	0.500	0.432	0.966 ^d^

Values are mean (standard deviation) or the number of appropriate participants. ^a^ Baseline vs. after 12 weeks of intervention, paired t-test. ^b^ Change in placebo vs. low- and high-dose after 12 weeks of intervention, unpaired t-test. ^c^ Baseline differences among the three groups. ^d^ One-way analysis of variance. ^e^ Chi-square test. ^*^ *P* < 0.05, ^**^ *P* < 0.01 vs. before the intervention or change in placebo.

## Supplementary Materials

The following are available online at https://www.mdpi.com/2072-6643/11/12/2844/s1, Table S1: Parameters before and after the intervention in each group.
