# Supplementary material for: Effects of Grape Seed Proanthocyanidin Extract on Vascular Endothelial Function in Participants with Prehypertension: A Randomized, Double-Blind, Placebo-Controlled Study
Source: Nutrients. 2019 Nov 20;11(12):2844. doi: 10.3390/nu11122844 (PMC6950399; doi:10.3390/nu11122844)
Supplement: Supplementary file 1 [file nutrients-11-02844-s001.pdf]

Table S1. Parameters before and after the intervention in each group

	Placebo (n = 10)							Low dose (n = 10)							High dose (n = 10)						
	0w	4w	8w	12w	P-value[a] 4w	P-value[a] 8w	P-value[a] 12w	0w	4w	8w	12w	P-value[a] 4w	P-value[a] 8w	P-value[a] 12w	0w	4w	8w	12w	P-value[a] 4w	P-value[a] 8w	P-value[a] 12w
Vascular functions																					
Resting vascular diameter, mm	3.82 (0.70)	3.79 (0.78)	3.87 (0.68)	3.69 (0.67)	0.736	0.722	0.182	3.68 (0.52)	3.69 (0.47)	3.73 (0.61)	3.75 (0.56)	0.912	0.577	0.492	3.85 (0.56)	4.00 (0.81)	3.61 (0.61)	3.64 (0.44)	0.184	0.185	0.236
Flow-mediated dilation (FMD), %	4.2 (1.5)	4.7 (2.1)	4.6 (2.0)	4.3 (1.7)	0.540	0.437	0.262	5.4 (1.6)	4.7 (2.1)	4.7 (1.5)	4.5 (3.1)	0.100	0.310	0.349	4.6 (1.3)	4.1 (1.4)	3.6 (2.4)	3.4 (2.4)	0.349	0.192	0.162
Baseline vascular diameter, mm	3.84 (0.69)	3.79 (0.84)	3.88 (0.67)	3.70 (0.70)	0.621	0.795	0.132	3.69 (0.54)	3.64 (0.44)	3.72 (0.58)	3.76 (0.55)	0.516	0.691	0.399	3.85 (0.58)	4.00 (0.84)	3.57 (0.61)	3.57 (0.46)	0.239	0.131	0.136
Flow-mediated dilation from baseline (FMDb), %	3.6 (1.9)	4.9 (2.1)	4.4 (1.5)	4.2 (2.0)	0.108	0.231	0.451	5.4 (2.4)	5.9 (2.1)	4.9 (1.9)	4.3 (2.5)	0.606	0.584	0.290	4.6 (2.2)	4.2 (2.1)	4.9 (1.8)	5.6 (2.7)	0.706	0.715	0.422
Intima-media thickness, mm	0.33 (0.08)	0.31 (0.05)	0.29 (0.05)	0.29 (0.08)	0.520	0.054	0.192	0.27 (0.04)	0.24 (0.04)	0.25 (0.04)	0.29 (0.06)	0.105	0.407	0.469	0.27 (0.05)	0.27 (0.03)	0.26 (0.05)	0.26 (0.04)	1.000	0.848	0.832
Wall thickness-to-vascular diameter ratio	0.09 (0.02)	0.08 (0.01)	0.08 (0.01)	0.08 (0.02)	0.840	0.102	0.355	0.08 (0.02)	0.07 (0.01)	0.07 (0.02)	0.08 (0.02)	0.136	0.372	0.741	0.07 (0.02)	0.07 (0.02)	0.07 (0.01)	0.07 (0.01)	0.817	0.673	0.726
Stiffness parameter β	23.1 (9.4)	23.0 (8.1)	24.7 (10.0)	19.8 (7.9)	0.853	0.525	0.301	25.5 (11.1)	24.7 (8.9)	26.3 (10.9)	21.7 (12.1)	0.827	0.768	0.340	28.0 (7.1)	25.4 (8.1)	20.4 (8.0)	20.6 (5.0)	0.428	0.036*	0.025*
Compliance ($\times 10^{-2}$), mm^2/Pa	10.2 (5.8)	9.1 (3.7)	9.1 (3.7)	10.3 (5.3)	0.394	0.469	0.953	8.9 (5.2)	8.8 (5.4)	8.7 (5.3)	10.7 (6.1)	0.981	0.876	0.355	7.4 (3.5)	10.5 (9.6)	9.9 (5.4)	8.9 (1.8)	0.268	0.048*	0.239
Distensibility ($\times 10^{-3}$) , Pa^{-1}	8.8 (3.9)	8.3 (3.6)	7.8 (3.4)	10.0 (5.6)	0.588	0.362	0.510	8.4 (5.0)	8.1 (4.2)	8.6 (6.3)	9.6 (5.2)	0.831	0.873	0.463	6.1 (1.6)	7.6 (3.7)	9.8 (5.3)	8.6 (1.7)	0.281	0.034*	0.004**
Incremental elastic modulus (Einc), kPa	1.6 (0.6)	1.8 (0.7)	2.0 (0.9)	1.7 (0.8)	0.564	0.158	0.931	2.1 (1.0)	2.2 (1.0)	2.5 (1.4)	1.9 (1.1)	0.798	0.386	0.309	2.53 (0.9)	2.2 (1.0)	1.7 (0.8)	1.7 (0.4)	0.451	0.061	0.018*
Pulse wave velocity (PWV), m/sec	12.3 (2.5)	12.7 (2.7)	13.0 (2.7)	11.6 (2.5)	0.604	0.423	0.417	12.9 (3.2)	12.7 (2.7)	13.1 (3.4)	12.2 (3.4)	0.836	0.811	0.515	14.1 (2.0)	13.0 (2.3)	11.7 (2.5)	11.7 (1.3)	0.243	0.016*	0.009**
Other cardiovascular parameters																					
Systolic blood pressure (SBP), mmHg	150.1 (11.1)	146.9 (14.9)	142.1 (16.6)	144.0 (17.0)	0.485	0.077	0.201	144.0 (18.1)	142.1 (18.1)	140.0 (17.7)	142.5 (17.1)	0.724	0.173	0.706	148 (13.0)	139.0 (9.7)	140.0 (11.1)	135.0 (12.6)	0.037*	0.066	0.028*
Diastolic blood pressure (DBP), mmHg	98.4 (10.2)	96.9 (10.6)	94.1 (11.3)	97.1 (8.4)	0.465	0.012*	0.489	95.9 (15.4)	93.0 (10.0)	92.5 (14.8)	94.3 (13.6)	0.359	0.010*	0.637	96.0 (6.9)	93.6 (5.5)	94.2 (7.1)	89.9 (7.9)	0.296	0.497	0.062
Heart rate, min^{-1}	69.9 (12.4)	69.4 (12.8)	67.8 (11.9)	68.8 (9.4)	0.785	0.354	0.685	72.6 (9.4)	70.7 (9.4)	71.3 (12.7)	71.8 (11.4)	0.624	0.771	0.837	67.0 (11.3)	65.8 (8.3)	64.9 (8.1)	69.9 (13.9)	0.681	0.276	0.375
Cardio-ankle vascular index	8.5 (0.7)	8.5 (0.8)	8.4 (0.9)	8.5 (0.7)	0.538	0.937	1.000	8.1 (1.3)	8.2 (1.1)	8.0 (1.1)	8.0 (1.1)	0.634	0.849	0.885	7.6 (1.1)	7.8 (1.1)	7.9 (1.1)	8.1 (0.9)	0.424	0.086	0.067
Ankle-brachial pressure index	1.12 (0.05)	1.12 (0.07)	1.13 (0.06)	1.13 (0.08)	0.917	0.663	0.686	1.10 (0.06)	1.12 (0.06)	1.11 (0.05)	1.12 (0.06)	0.271	0.439	0.336	1.13 (0.08)	1.13 (0.07)	1.14 (0.08)	1.18 (0.06)	1.000	0.524	0.135
High-density lipoprotein cholesterol, mg/dL	63.3 (19.4)	65.7 (15.3)	64.4 (15.7)	61.7 (10.0)	0.510	0.766	0.647	70.6 (16.0)	68.3 (16.7)	71.1 (15.9)	66.8 (15.7)	0.174	0.859	0.244	77.9 (14.7)	78.4 (14.5)	80.1 (14.6)	75.6 (11.7)	0.810	0.256	0.375
Low-density lipoprotein cholesterol, mg/dL	111.0 (25.4)	118.7 (24.7)	105.4 (27.7)	115.6 (25.7)	0.221	0.153	0.301	128.2 (37.1)	128.6 (41.5)	127.2 (36.6)	126.1 (35.9)	0.947	0.776	0.694	110.8 (32.5)	114.0 (26.6)	113.6 (28.7)	112.1 (21.2)	0.556	0.592	0.835
Oxidized low-density lipoprotein cholesterol, U/L	116.6 (47.2)	114.8 (35.8)	96.0 (49.6)	97.7 (38.2)	0.874	0.143	0.262	115.8 (45.6)	122.1 (44.5)	109.5 (41.0)	110.1 (48.4)	0.525	0.586	0.705	106.2 (34.8)	104.6 (35.9)	110.5 (40.8)	113.3 (29.5)	0.879	0.760	0.507
Triglyceride, mg/dL	99.9 (55.2)	19.9 (101.2)	129.7 (124.0)	114.9 (46.8)	0.236	0.233	0.234	87.9 (55.3)	125.0 (73.3)	97.3 (44.0)	149.5 (125.6)	0.067	0.535	0.096	103.5 (71.7)	110.3 (67.0)	121.1 (71.4)	121.3 (74.4)	0.534	0.142	0.116
Total cholesterol, mg/dL	192.5 (26.5)	206.0 (21.4)	192.2 (24.0)	196.2 (22.8)	0.036*	0.946	0.459	219.1 (31.8)	221.3 (32.7)	214.8 (28.4)	219.1 (32.1)	0.484	0.244	1.000	207.5 (41.1)	212.6 (35.2)	212.1 (37.9)	207.4 (33.1)	0.356	0.422	0.987
Body composition																					
Height, cm	158.7 (11.6)	158.7 (11.6)	158.7 (11.5)	158.8 (11.4)	0.994	0.997	0.986	159.1 (8.5)	158.9 (8.5)	159.0 (8.5)	158.9 (8.4)	0.975	0.986	0.963	159.6 (10.5)	159.3 (10.4)	159.3 (10.2)	159.4 (10.4)	0.951	0.958	0.961
Weight, kg	59.4 (13.1)	59.4(13.0)	59.7 (13.1)	59.2 (13.1)	0.739	0.524	0.524	58.9 (8.6)	59.4 (8.5)	59.5 (8.5)	59.7 (8.6)	0.123	0.131	0.076	57.6 (13.7)	57.8 (13.6)	57.6 (13.4)	57.5 (12.6)	0.402	0.853	0.844
Body mass index, kg/m^2	23.4 (3.4)	23.4 (3.4)	23.5 (3.4)	23.3 (3.5)	0.521	0.453	0.311	23.4 (3.7)	23.6 (3.8)	23.6 (3.9)	23.7 (3.8)	0.053	0.090	0.050*	22.5 (3.9)	22.6 (3.9)	22.5 (4.0)	22.5 (3.7)	0.129	0.563	0.751
Fat mass, kg	17.5 (7.1)	17.2 (7.1)	17.5 (7.3)	17.1 (7.4)	0.237	0.943	0.175	17.9 (7.2)	18.3 (7.6)	18.1 (7.8)	17.9 (7.6)	0.232	0.540	0.969	15.4 (7.9)	15.5 (7.9)	15.4 (8.1)	15.5 (7.4)	0.487	0.972	0.744
Lean body mass, kg	41.9 (9.7)	42.2 (9.8)	42.2 (9.4)	42.1 (9.4)	0.058	0.350	0.050	41.0 (5.1)	41.2 (4.9)	41.4 (5.2)	41.8 (5.7)	0.610	0.177	0.744	42.2 (9.2)	42.3 (9.3)	42.2 (9.1)	42.0 (8.9)	0.880	0.780	0.444
Muscle mass, kg	39.6 (9.2)	39.9 (9.3)	39.8 (8.9)	39.8 (8.9)	0.051	0.368	0.331	38.7 (4.9)	38.8 (4.7)	39.0 (4.9)	39.5 (5.4)	0.608	0.176	0.047*	39.9 (8.8)	39.9 (8.8)	39.8 (8.7)	39.7 (8.5)	0.993	0.987	0.961
Water mass, kg	30.0 (5.8)	30.3 (6.0)	30.3 (5.6)	30.1 (5.6)	0.103	0.255	0.512	29.5 (3.0)	29.8 (2.9)	30.2 (3.0)	30.6 (3.7)	0.334	0.029*	0.035[e]	29.7 (5.2)	29.9 (5.3)	29.8 (5.3)	29.6 (5.0)	0.490	0.435	0.771
Basal metabolic rate, MJ/day	5.04 (1.06)	5.07 (1.07)	5.07 (1.03)	5.05 (1.04)	0.074	0.415	0.574	4.95 (0.53)	4.97 (0.49)	5.00 (0.51)	5.04 (0.57)	0.437	0.106	0.027[e]	5.05 (1.04)	5.05 (1.03)	5.04 (1.01)	5.02 (0.98)	0.803	0.703	0.500

Values are mean (standard deviation).

[a]Baseline vs. after 4, 8, and 12 weeks of intervention, paired t-test.

*P < 0.05, **P < 0.01 vs. before the intervention.
